# Neuroprotective effects of isoflavone-enriched soybean leaves (*Glycine Max*) on scopolamine-induced memory deficits in C57BL/6 mice via antioxidative mechanisms

**DOI:** 10.3389/fnagi.2026.1787268

**Published:** 2026-04-10

**Authors:** Young Ju Do, Seo Young Kim, Younghoon Go, Yong Hyun Lee, Ki Hun Park, Tae Woo Oh

**Affiliations:** 1 Department of Oriental Medicine Research Division, Korea Institute of Oriental Medicine (KIOM), Daejeon, Republic of Korea; 2 University of Science and Technology (UST), Korean Convergence Medicine Major KIOM, Daejeon, Republic of Korea; 3 Korean Medicine (KM) Application Center, Korea Institute of Oriental Medicine (KIOM), Daegu, Republic of Korea; 4 Division of Applied Life Science (BK21 four), Institute of Agricultural and Life Science (IALS), Gyeongsang National University, Jinju, Republic of Korea

**Keywords:** isoflavone-enriched soybean leaves (IESLs), scopolamine, cognitive impairment, neuroprotection, oxidative stress, BDNF

## Abstract

**Introduction:**

This study aimed to investigate the neuroprotective and memory-enhancing effects of isoflavone-enriched soybean leaves (IESLs) against scopolamine-induced cognitive impairment. While soybean leaves (SLs) are naturally rich in kaempferol glycosides and soyasaponins, IESLs are characterized by significantly elevated isoflavone levels through metabolite farming.

**Methods:**

To assess the efficacy of IESLs, C57BL/6 mice were orally administered IESLs (6.25 and 18.8 mg isoflavones/kg/day) for 5 weeks, with scopolamine (1 mg/kg, i.p.) administered to induce memory impairment. Spatial learning and memory functions were evaluated using the Morris water maze and passive avoidance tests.

**Results:**

Scopolamine-induced behavioral deficits were significantly restored by IESLs administration in a dose-dependent manner. Mechanistically, IESLs treatment reduced the intracellular accumulation of reactive oxygen species and enhanced the antioxidant defense system. Furthermore, IESLs significantly upregulated the expression of brain-derived neurotrophic factor (BDNF) and activated the cAMP response element-binding protein (CREB) via phosphorylation. Histological analysis also revealed that IESLs suppressed the activation of astrocytes and microglia, thereby attenuating neuroinflammation.

**Discussion:**

These findings suggest that IESLs effectively alleviate cognitive impairment by strengthening antioxidant defenses and activating the CREB-BDNF signaling pathway. Consequently, IESLs may serve as a promising therapeutic or preventive candidate for amnesia and neurodegenerative diseases.

## Introduction

The rapid aging of the population has led to a growing proportion of elderly people with dementia, posing a serious social problem. Dementia gradually leads to cognitive dysfunction, including impairments in intelligence, learning ability, memory, problem-solving, attention, concentration, and judgment. It may also cause behavioral and psychological symptoms such as insomnia, irritability, anxiety, depression, impulsivity, delusions, and hallucinations—all without impairment of consciousness (Tagai et al., 2020). Senile dementia is categorized into cerebrovascular dementia and Alzheimer’s disease (AD). Since the treatment of cerebrovascular disease primarily serves as the foundation, drugs that improve cerebral blood flow, protect brain cells, and promote their recovery are commonly used (Kuang et al., 2021). However, the cause of AD remains unclear, and no effective treatment has been established. The main treatments include cholinesterase inhibitors, which are drugs that improve brain metabolism by affecting neurotransmitter systems. Antioxidants are also used as a supplementary treatment (Maliszewska-Cyna et al., 2017).

Acetylcholine (ACh) is a major neurotransmitter and modulator of the nervous system. It plays an important role in cognitive functions, such as learning and memory, as well as at the neuromuscular junction and in the parasympathetic nervous system. Increased activity of enzymes such as acetylcholinesterase (AChE) and butyrylcholinesterase (BuChE) in the brain breaks down the neurotransmitter acetylcholine into choline and acetate (Talesa, 2001), reducing cholinergic signaling and impairing memory and cognitive function (Kasa et al., 1997; Talesa, 2001). Scopolamine, a muscarinic cholinergic receptor antagonist, induces cholinergic dysfunction and oxidative stress in the brain, leading to memory impairment. This drug is primarily used in animal models of cholinergic memory impairment for verification (Konar et al., 2019). Although the scopolamine model does not reproduce progressive neuropathological hallmarks of AD, such as amyloid-*β* deposition or tau pathology, it is widely used as a pharmacological model of Alzheimer’s -like cognitive dysfunction based on the cholinergic hypothesis of AD (Klinkenberg and Blokland, 2010). In addition to cholinergic dysfunction, scopolamine has been reported to induce oxidative stress and suppress CREB phosphorylation and BDNF expression in the hippocampus, mechanisms that are closely associated with synaptic plasticity and early cognitive decline. Therefore, this model is suitable for investigating therapeutic strategies targeting cholinergic and oxidative stress-related cognitive impairment. Therapeutic agents for dementia, such as donepezil, rivastigmine, and galantamine, are typically used for moderate-to-severe AD (Ghai et al., 2020). However, these drugs are primarily used to alleviate symptoms. Given the severe side effects of acetylcholine-degrading enzyme inhibitors, developing drugs to overcome these limitations is an urgent priority.

Plants produce secondary metabolites as a survival response to environmental conditions. Since these metabolites are essential for plant survival, they are also beneficial for humans who consume plants. This phenomenon is known as xenohormesis (Hooper et al., 2010). Recently, some studies have explored the production of bioactive metabolites by modulating the external environment (Yuk et al., 2016; Kim et al., 2023; Lee et al., 2024; Im et al., 2025). Soybean leaves (SLs) are consumed in some parts of Asian countries and are also permitted as an edible ingredient by the Korean Food and Drug Administration. SLs have been shown to suppress hepatic lipid accumulation, fat oxidation, and inflammation. SLs are naturally rich in kaempferol glycosides and soyasaponins but contain only trace amounts of isoflavones. Recently, IESLs were prepared through metabolite farming using ethylene treatment, which selectively increased phytoestrogens such as daidzin, genistin, malonyl daidzin, and malonyl genistin. Isoflavones are the most abundant metabolites in IESLs, along with kaempferols and soyasaponins, all of which are naturally present in SLs (Yuk et al., 2016). The pharmacological effects of IESLs include preventing obesity by improving fatty acid oxidation in an ovariectomized (OVX) animal model (Xie et al., 2018). In studies related to brain diseases, IESLs have been reported to alleviate cognitive impairment and regulate PI3K/Akt signaling in the hippocampus of ovariectomized Sprague–Dawley rats (Yoo et al., 2022). In addition, IESLs suppress high-fat diet–induced fatty liver in mice (Kim et al., 2017). Genistein and daidzein participate in the BDNF-extracellular signal-TRK signaling pathway in the hippocampus, promoting neuronal survival and proliferation (Pan et al., 2012). Moreover, S-equol, a soybean isoflavone metabolite, has been reported to potentially mitigate cognitive decline and dementia linked to gut microbiota (Sekikawa et al., 2022).

Notably, accumulating evidence suggests that scopolamine administration not only induces cholinergic dysfunction but also disrupts hippocampal BDNF expression and CREB phosphorylation, while increasing oxidative stress markers (Abuelezz and Hendawy, 2023). Because oxidative imbalance and impaired neurotrophic signaling are closely associated with cognitive decline, this model provides a suitable platform to investigate antioxidant and BDNF-related mechanisms of neuroprotection. Despite these findings, many natural extracts still face limitations due to low concentrations of active metabolites. To overcome this, we utilized a ‘metabolite farming’ approach to increase neuroprotective isoflavones in soybean leaves by 140-fold. While previous studies often focused on single-target effects, this research fills the gap by demonstrating how these enriched IESLs simultaneously regulate cholinergic function, oxidative stress, and the BDNF-CREB pathway. This multi-target approach provides a more comprehensive strategy for addressing early-stage cognitive decline.

We hypothesized that IESLs alleviate scopolamine-induced cognitive impairment by strengthening the antioxidant defense system and activating the CREB–BDNF signaling pathway. To test this, we evaluated the antioxidant potential of IESLs and systematically investigated their efficacy in restoring memory and cognitive function. Using a scopolamine-induced memory-impaired mouse model, we performed behavioral assays to assess cognitive recovery. Furthermore, the underlying molecular mechanisms were elucidated by analyzing the BDNF/TrkB and apoptotic pathways.

## Materials and methods

### Preparation of IESLs

IESLs, synthesized using the method of Yuk et al. (2016) were provided by Ki Hun Park’s lab at Gyeongsang National University (Jinju, Korea; Xie et al., 2018). IESLs were prepared using metabolite farming approaches to significantly increase the accumulation of isoflavone derivatives in soybean leaves. Soybean plants were cultivated in the field for approximately 2 months until reaching the R3 growth stage, and more than 100 plants were harvested for the preparation of the extract. At this stage, the plants were uprooted and transferred to pots containing nutrient solution. The plants were subjected to ethylene treatment in an air-sealed chamber. Ethylene gas was applied at a concentration of 2,500 ppm for 24 h under controlled conditions. After ethylene treatment, the soybean leaves were harvested, dried, and separated from other plant tissues. The dried leaves (20 kg) were extracted with hot water at 100 °C for 6 h, and the extract was subsequently concentrated to obtain the final material used in the animal experiments. The extract obtained from ethylene-treated soybean leaves is hereafter referred to as IESLs. The chemical profile of the extracts was confirmed using LC-Q-TOF/MS and HPLC analyses, as shown in Supplementary Figures S1, S2.

### Animal and treatment study

**Figure 1:**
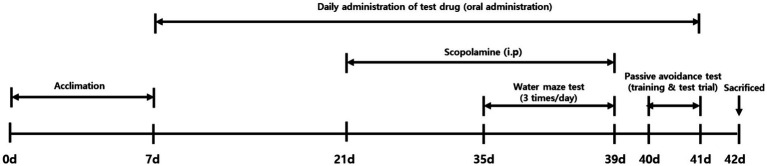
Schematic of the experimental design.

Male C57BL/6 mice (8 weeks old) were purchased from DooYeol Biotech (Seoul, Korea). After a one-week adaptation period, the animals were divided into five groups: vehicle-treated control (Control, *n* = 10); scopolamine-treated vehicle (Sco, *n* = 10); scopolamine-treated with IESLs (6.25 mg isoflavones/kg/day, low dose; Sco + Low, *n* = 10); scopolamine-treated with IESLs (18.8 mg isoflavones/kg/day, high dose; Sco + High, *n* = 10); scopolamine-treated with the positive control drug, donepezil (5 mg/kg/day; Sco + Done, *n* = 10). IESLs were administered orally once daily for 5 weeks. The doses of IESLs were determined based on the Human Equivalent Dose (HED) derived from the recommended daily intake of soy isoflavones (24–28 mg/60 kg/day) for adults, as specified in the Health Functional Food Code. Using a body surface area-based conversion method, the human reference dose of 27 mg/60 kg (0.45 mg/kg) was translated to a mouse dose of approximately 6.25 mg/kg. This dose was established as the low-dose (Sco + Low), while a threefold higher dose (18.8 mg/kg) was selected as the high-dose (Sco + High) to evaluate dose-dependent neuroprotective effects. During the experiment, mice were housed in a 12-h light/dark cycle environment, and the same diet was provided ad libitum to both the experimental and control groups. All animal procedures were approved by the Korea Institute of Oriental Medicine-Institutional Animal Care and Use Committee (KIOM-IACUC; reference number: 22–041). The experimental schedule is shown in Figure 1.

After a 1-week adaptation period, C57BL/6 mice were orally administered vehicle (saline), IESLs, or donepezil (5 mg/kg) for 5 weeks. Starting on day 21, they received scopolamine (1 mg/kg) for 14 days treated intraperitoneally with vehicle (saline). The Morris water maze was performed on days 35–39 to assess memory function. All mice were euthanized on day 42 under deep anesthesia induced by isoflurane inhalation (3–4% for induction, followed by 1.5–2% for maintenance in oxygen). After confirmation of loss of reflexes, the animals were sacrificed by cervical dislocation. The hippocampus was subsequently collected for further biochemical analysis. P. O.; per os (by daily, orally), I. P.; intraperitoneal.

### Measurement of body weight

After confirming the overall health and behavioral status of all mice prior to physiologic testing, body weight was measured and recorded weekly, every Monday. Body weight was measured on day 1 of drug administration and at the end of the experiment to compare the weight gain ratio among groups.

### Morris water maze test

The MWM test was conducted to assess spatial memory and learning abilities in all mice. The water maze consisted of a 1.2-m-diameter rubber pool filled with opaque water (reverse osmosis water mixed with white paint) and a 10-cm-diameter hidden platform in the northeast (NE) quadrant. An overhead video camera connected to the SMART video tracking system (Panlab, Barcelona, Spain) was used to track and record the mice’s swimming behavior. The mice underwent four habituation training sessions on day 0. The platform was visible (2 cm above the water surface) in undyed water. Test trials were conducted over five consecutive days (day 1 to day 5). These behavioral tests were conducted in accordance with previous research (Bae et al., 2024).

### Passive avoidance test

The PA test was conducted using a Shuttle Box Avoidance Basic Test Package (Med Associates Inc., Fairfax, VT, USA). The apparatus consisted of two connected rooms—one illuminated and one darkened—linked by an automatic door. The darkened room was equipped with a grid floor through which an electric shock (0.5 mA) could be delivered. Step-through latency times (maximum cutoff: 2 min) were recorded as mice moved from the illuminated room to the darkened room. The PA test was conducted as previously described (Bae et al., 2024).

### Tissue processing

The brain was removed, including the cerebellum and brainstem, and other tissues were collected. The tissues were fixed in 4% paraformaldehyde in PBS (pH 7.4) for 24 h at room temperature (*n* ≥ 3 per group) for subsequent H&E and immunohistochemical staining. The prepared tissues were fixed for over 24 h before dehydration using a graded alcohol series. The samples were sequentially soaked in alcohol-benzene and xylene before paraffin embedding. The wax-soaked tissues were cut into 5-μm-thick slices. Finally, the finished pieces were stored at room temperature.

### Hematoxylin and eosin staining

Wax-embedded brain tissue sections were prepared. Initially, tissue sections were dewaxed with xylene (twice, 20 min each) followed by a graded ethanol series (100%, twice, 10 min each; then 95, 90, 80, 70, and 50%, once each for 5 min). After washing in water, the wax slices were stained with hematoxylin and eosin. Finally, entire pieces were examined under an optical microscope (Olympus, Hamburg, Germany).

### Immunohistochemical staining

Fixed brain sections were deparaffinized in xylene and rehydrated through a graded ethanol series. In brief, brain section slices were immersed in xylene and rehydrated through a graded ethanol series (100, 95, 90, 80, 70, and 50%). To block endogenous peroxidase, sections were incubated in 3% H₂O₂ for 30 min, followed by incubation with 5% normal goat serum for 1 h. After blocking, the sections were incubated overnight at 4 °C with primary antibodies (anti-NeuN and anti-GFAP; Novus Biologicals, Minneapolis, MN, USA), followed by incubation with a peroxidase-conjugated secondary antibody for 1 h. The samples were washed again with PBS, followed by staining with 3,3′-diaminobenzidine tetrahydrochloride (DAB). Next, hematoxylin was used to re-stain, and neutral resin was used to mount the brain sections. Finally, images of the stained sections were acquired using an optical microscope (Olympus, Hamburg, Germany). Representative images were obtained from the hippocampus. Quantitative analysis was performed using ImageJ software. For each animal, three non-overlapping sections from the hippocampus were analyzed, and the mean value was used for statistical comparison. All image acquisition settings and analysis parameters were kept constant across groups. The investigator performing the quantification was blinded to the treatment groups to minimize bias. The sample size was determined based on established protocols in scopolamine-induced models to ensure adequate statistical power for detecting differences in cognitive and histological parameters.

### Terminal deoxynucleotidyl-transferase–mediated dUTP nick end labeling (TUNEL) staining

After all experiments were completed, the fixed brains were embedded in paraffin blocks and sectioned at 5 μm. Apoptotic cells in the cortex were detected using the terminal deoxynucleotidyl transferase dUTP nick end labeling (TUNEL) method. TUNEL staining was performed six times following the manufacturer’s protocol (R&D, Germany). TUNEL-positive cells exhibited green fluorescence and were quantified using fluorescence microscopy (Olympus BX53; Olympus Corporation, Hamburg, Germany) at magnifications of ×100 and ×400. For each section, five fields from the ischemic cortex were examined. The average percentage of TUNEL-positive cells relative to the total cell count was determined. Nuclei were stained with DAPI.

### Western blot analysis

Proteins were extracted from mouse tissues in the scopolamine-induced cognitive impairment model using RIPA lysis buffer containing protease and phosphatase inhibitor cocktails (Roche, Basel, Switzerland). The protein was separated via centrifugation at 12,000 rpm for 20 min. The concentration of each isolated protein was quantified using a protein assay solution. Then, 30 μg of protein was mixed with 5 × sample buffer and separated through 8–15% SDS-PAGE. Proteins from the isolated gels were transferred to NC membranes, and each membrane was blocked with 5% BSA at room temperature for 1 h. Primary antibodies targeting markers of differentiation, cell death (Bax, Bcl-2, caspase-3, cleaved-caspase-3, and PARP), and suppression of trophic factors (BDNF, CREB, pCREB, and TrkB) were added to the membrane and incubated overnight at 4 °C. The membrane was then washed three times with TBS containing 0.05% Tween-20. After incubating the membrane with the HRP-conjugated anti-IgG antibody at room temperature for 1 h, it was washed three times with TBS (1 × TTBS) containing 0.05% Tween-20. Detection was then performed using the ChemiDoc™ Touch Imaging System (Bio-Rad, California, United States) with ECL solution.

### Biochemical assays

The concentrations of malondialdehyde (MDA) and glutathione (GSH), as well as the activities of catalase and superoxide dismutase (SOD), were measured in homogenized brain tissue using the following assay kits: a Lipid Peroxidation (MDA) Colorimetric Assay Kit (Abcam, Cambridge, United Kingdom) and catalase, SOD, and GSH assay kits (Cayman Chemical, Michigan, United States). The protein concentration was quantified using a BCA kit.

### Statistical analysis

All experimental data are presented as the mean ± standard deviation (SD) or mean ± standard error of the mean (SEM) from at least three independent experiments. For the Morris water maze (MWM) training sessions, escape latency was analyzed using a two-way repeated-measures ANOVA to account for the correlated nature of repeated behavioral measurements over five consecutive days, with ‘Day’ as the within-subject factor and ‘Treatment’ as the between-subject factor. Other behavioral and biochemical data involving more than two groups were analyzed using one-way ANOVA. Additionally, mean swimming speeds were evaluated using one-way ANOVA to confirm the absence of motor impairment as a confounding variable. Following ANOVA, Dunnett’s or Tukey’s multiple comparison tests were performed for post-hoc analysis to determine statistically significant differences between specific groups. For comparisons between only two groups, Student’s t-test was employed. All statistical analyses were conducted using GraphPad Prism version 10 (GraphPad Software, San Diego, CA, United States). A value of *p* < 0.05 was considered significant (*p < 0.05, **p < 0.01, and ***p < 0.001) in GraphPad Prism version 10 for Windows (GraphPad Software, San Diego, CA, United States).

## Results

### IESLs

IESLs were prepared in an air-tight chamber using ethylene treatment, following a previously established method (Ban et al., 2020). Most bioactive metabolites in the 80% ethanol extract of IESLs were identified using LC–ESI-Q–TOF/MS (Supplementary Figure S1). They are classified into three groups: seven kaempferol glycosides, seven isoflavone glycosides, and four soy’ as a ponins. Supplementary Figure S2 compares HPLC chromatograms at 254 nm between IESLs and normal SLs. SLs have kaempferol glycosides as their main active metabolites, whereas IESLs contain not only kaempferol glycosides but also a significant amount of isoflavone glycosides, including daidzin, genistin, malonyldaidzin, and malonylgenistin. The four major isoflavones (daidzin, genistin, malonyldaidzin, and malonylgenistin) were quantitatively analyzed using external standard calibration curves. Calibration curves were generated using authentic standards at the following concentration ranges: 7.8125–250 μg mL^−1^ for daidzin and genistin, and 62.5–1,000 μg mL^−1^ for malonyldaidzin and malonylgenistin. The calibration equations were as follows: daidzin, y = 34.5679x + 154.6485 (R^2^ = 0.9998); genistin, y = 39.4832x + 141.4170 (R^2^ = 0.9986); malonyldaidzin, y = 14.2559x + 143.6829 (R^2^ = 0.9998); malonylgenistin, y = 15.2266x + 55.7242 (R^2^ = 0.9999). The high correlation coefficients (R^2^ > 0.998) indicate excellent linearity of the calibration curves for quantitative analysis. Quantitative analysis revealed that IESLs contain 140 times more isoflavones than SLs while maintaining nearly equivalent levels of kaempferols. In particular, the isoflavones in IESLs are in glycoside form, which has a much higher (2–3-fold) bioavailability than the aglycone form (Rufer et al., 2008). Given the advantages described above, IESLs may exhibit a specific physiological effect.

### Effect of IESLs on scopolamine-induced spatial memory impairment in mice

**Figure 2:**
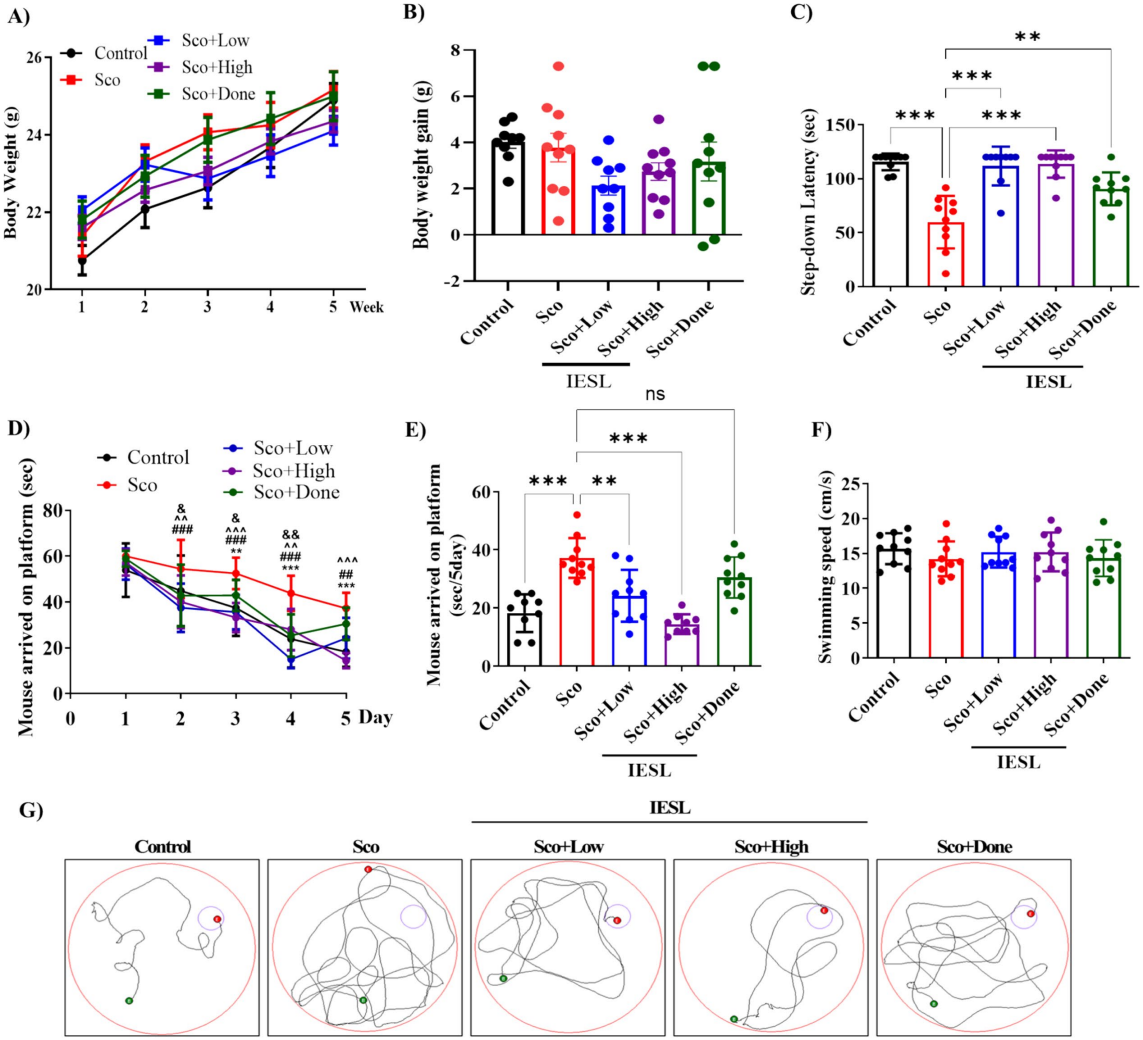
Preventive effects of IESLs on scopolamine-induced cognitive and memory dysfunction in C57BL/6 mice. **(A)** Body weight, **(B)** body weight gain, **(C)** the performance of the mice in the step-down passive avoidance test, **(D)** arrived on platform by day; ^**^p < 0.01 and ^***^p < 0.001 (Control vs. Sco), ^##^*p* < 0.01 and ^###^*p* < 0.001 (Sco vs. Sco + Low), ^^*p* < 0.01 and ^^^ *p* < 0.001 (Sco vs. Sco + High), ^&^*p* < 0.05 and ^&&^*p* < 0.01 (Veh vs. Sco + Done). **(E)** Mice arriving at the platform in a Morris water maze (MWM) test **(F)** the swimming speed of each group in 5-day MWM test (Two-way Repeated Measured ANOVA with Tukey’s *post-hoc* test) and **(G)** representative swimming paths, Values are expressed as the means ± SEMs of three independent experiments. ^**^p < 0.01 and ^***^p < 0.001 vs. Control or Sco. *n* = 10 mice per group.

Body weight changes in 5-week-old mice—control (Control), scopolamine-treated (Sco), IESLs-treated (Sco + Low and Sco + High), and positive control-treated (Sco + Done)—are shown in Figures 2A,B. There was no significant difference in body weight among the all groups at the end of the study.

Body weight changes during the 5-week administration period showed no significant differences among groups (Figures 2A,B). Consistently, total body weight change did not differ significantly between groups (Figure 2B). These results suggest that none of the treatments induced negative changes in body weight, indicating no apparent systemic burden under the experimental conditions.

To investigate the effect of IESLs on scopolamine-induced memory impairment, we conducted a water maze experiment to assess spatial perception, short-term memory, and long-term memory. Scopolamine acts as an acetylcholine receptor antagonist and impairs memory (Asadi Rizi et al., 2024). The results of the passive avoidance test revealed that the control group remained in the bright room for 115.5 ± 7.77 s, the Sco group treated with scopolamine remained in the bright room for 59.75 ± 24.31 s, whereas the scopolamine-only (Sco) group showed a significant decrease. In the experimental groups (Sco + Low, Sco + High) treated with IESLs, the time increased in a concentration-dependent manner to 111.7 ± 17.64 s and 113.5 ± 12.56 s, respectively. In comparison with the Sco + Done group showed a time of 90.6 ± 15.20 s (Figure 2C). Memory was assessed using the mean arrival time (time to reach the platform) as an index, reflecting how long the experimental animals took to recognize environmental cues and locate the hidden escape platform. Consequently, the control group and the scopolamine-only (Sco) group exhibited statistically significant differences on the second, fourth, and fifth days of training (Figure 2D). In the control group, time spent in the escape zone decreased significantly as the training period progressed. Meanwhile, animals treated with scopolamine alone (intraperitoneally) rarely or delayedly entered the escape zone during the training duration (Figure 2D). In contrast, the groups co-administered IESLs (Sco + Low, Sco + High) showed a gradually improved over 1 to 5 days (with shorter times) mean escape time than the scopolamine-only group (Sco), with performance on the last day (5 day) recovered to a level similar to that of the control group (Figure 2D). On the final day (day 5), the mean escape times were 37.2 ± 6.82 s for the Sco group, 24.2 ± 8.90 s for the Sco + Low group, and 14.4 ± 3.35 s for the Sco + High group. The difference of 22.8 s between the scopolamine and combination donepezil (Sco + Done) groups was statistically significant (Figure 2E). In both the control group and the IESLs-administered experimental group (Sco + Low, Sco + High), a stable decline was observed from the 2 day onward in the graph, indicating long-term memory formation over the subsequent four-day experimental period. There were no significant differences in mean swimming speed among the groups (Control: 15 ± 2.20 cm/s, Sco: 14 ± 2.49 cm/s, Sco + Low: 15 ± 2.20 cm/s, Sco + High: 15 ± 2.76 cm/s, Sco + Done: 14 ± 2.61 cm/s), suggesting that IESLs treatment did not affect the locomotor activity of the mice (Figure 2F). Figure 2G shows the representative routes taken by experimental animals in each group to reach the platform during the five-day training period.

### Effect of IESLs on histological changes in the brains of scopolamine-treated mice

**Figure 3:**
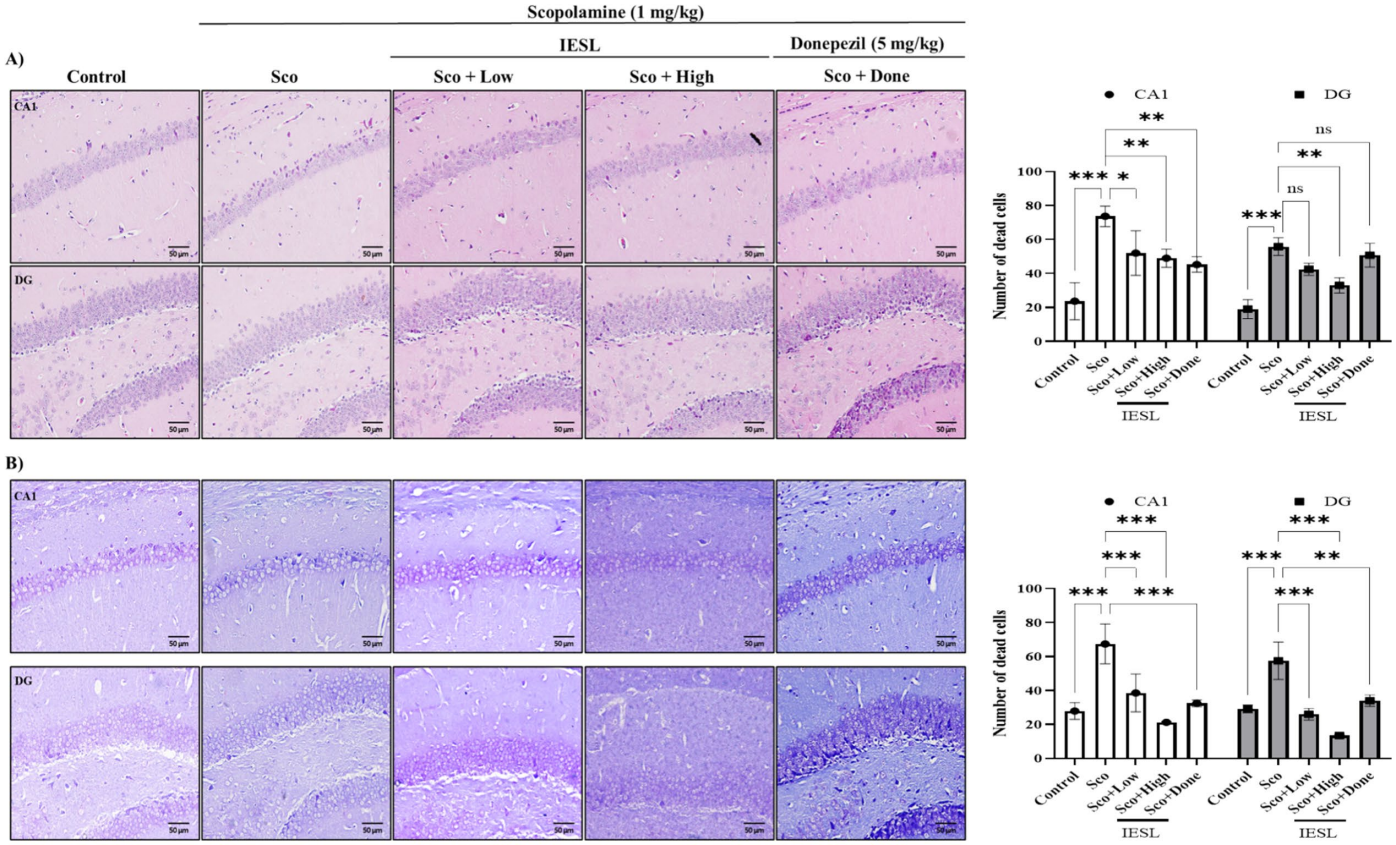
Effects of IESLs on neuronal damage in scopolamine-induced mice. **(A)** Representative images of H&E staining in mice hippocampus (CA1, upper), and dentate gyrus (DG, lower). **(B)** Representative images of Nissl staining in mice hippocampus (CA1, upper), and dentate gyrus (DG, lower). The photograph is a representative image of three different tissues and original magnification ×400. Mean ± SEM from *n* = 3 per group. ^*^p < 0.05, ^**^p < 0.01 and ^***^p < 0.001 vs. Control or Sco.

Scopolamine, a muscarinic cholinergic receptor antagonist, damages and induces atrophy in both brain nerves and hippocampal and cerebral cortical neurons in mice (Asadi Rizi et al., 2024) Therefore, in this study, the brains were removed immediately after mouse dissection and examined under an optical microscope using H&E and Nissl staining (Figure 3). As a result, in scopolamine-damaged brain tissue, hippocampal pyramidal neurons showed morphological deformities (ghost cells), including nuclear fragmentation, clumping, and pyknosis due to ischemic damage. In addition, cell counts decreased, and the surrounding tissue density was reduced. However, in the experimental group that received IESLs, the pyramidal neurons in the hippocampal CA1 region had a typical morphology, as most remained intact (Figure 3A). Meanwhile, Nissl staining was used to confirm the presence of Nissl bodies in hippocampal neurons. Consistent with the H&E staining results, hippocampal neurons in the Sco group (scopolamine-treated) were highly condensed compared with those in the control group, which showed round nuclei and preserved structural integrity. Notably, the Sco group showed a significant reduction in neuronal count, although intraperitoneal scopolamine administration resulted in milder damage (Figure 3B).

### Effect of IESLs on the activation of astrocytes and microglia in the brains of scopolamine-induced mice

**Figure 4:**
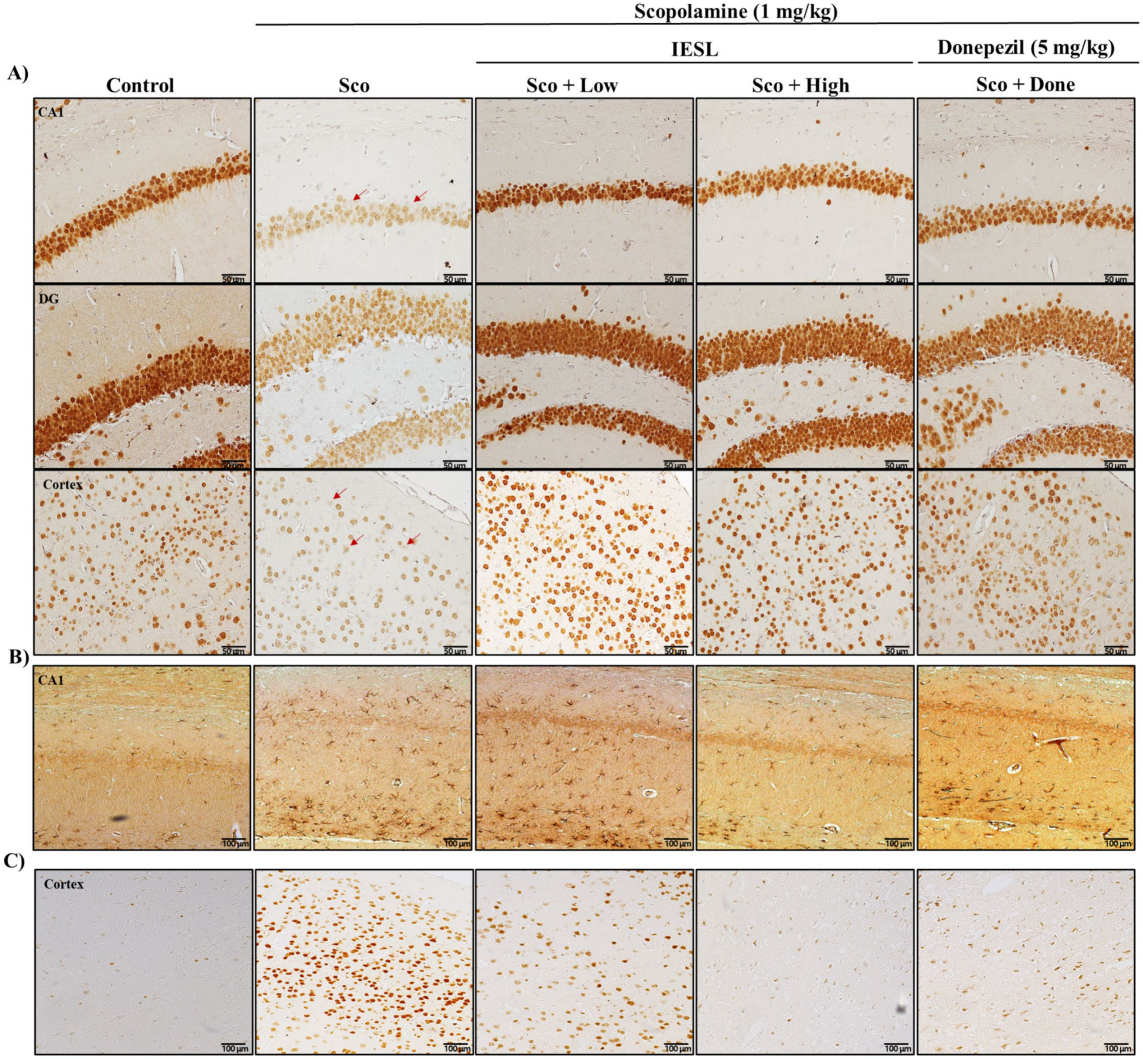
Effects of IESLs on the activation of glial cells in the brain of scopolamine-induced mice. Brain tissues were immune-stained by **(A)** anti-NeuN, **(B)** anti-GFAP, and **(C)** anti-CD11b antibodies. **(A)** The morphological changes of neuronal cells (original magnification ×400), **(B)** astrocytes, and **(C)** microglia were observed in the penumbra of ischemic rats by microscope (original magnification ×200). The photograph is a representative image of three different tissues.

To investigate the effect of IESLs on the activation of neuronal and glial cells in the brain, we assessed morphological changes in astrocytes, microglia, and neurons using immunohistochemistry. Scopolamine induced the loss of NeuN-positive neurons (Figure 4A) and activated inflammatory cells, including GFAP-positive astrocytes (Figure 4B) and CD11b-positive microglia (Figure 4C). Neuroglial cells—including astrocytes and microglial cells—play a key role in inflammation. When neurons are damaged, they adopt a reactive morphology. We assessed the effects of scopolamine on neurons using anti-NeuN immunoreactivity. The scopolamine-treated (Sco) group showed a reduction in NeuN-positive neurons and decreased expression in the CA1, DG, and cortical regions. In contrast, the IESLs group showed a significant increase in NeuN-positive neurons without morphological changes (Figure 4A). Reactive astrocytes weaken synaptic support, release inflammatory and neurotoxic factors, and induce neuronal death and brain atrophy, ultimately leading to severe dementia. Reducing reactive astrocyte abundance is important for normal neuronal function (Ahmad et al., 2025). Therefore, we determined anti-GFAP immunoreactivity (an astrocyte marker) to evaluate the effect of IESLs on astrocyte changes. Scopolamine treatment increased GFAP-labeled reactive astrocytes in the CA1 region. These results indicate that astrocytes transformed into reactive forms due to scopolamine-induced brain changes. To provide a quantitative assessment of glial activation, the number of GFAP-positive reactive astrocytes and CD11b-positive activated microglia was determined using ImageJ software, following the standardized protocols described in the Methods section. Scopolamine treatment significantly increased the density of GFAP-labeled reactive astrocytes in the CA1 region, indicating a shift toward a reactive morphology. However, the number of these reactive astrocytes was significantly lower in the IESLs-treated group. Similarly, while prominent CD11b-positive microglial activation was observed in the scopolamine-treated group, IESLs administration effectively suppressed this neuroinflammatory response. Although CD11b was utilized here as an established marker for activated microglia in this model, these findings collectively demonstrate that IESLs alleviate scopolamine-induced neurotoxicity by modulating glial overactivation.

### Changes in neurotrophic factors and activators in the brains of scopolamine-induced mice

**Figure 5:**
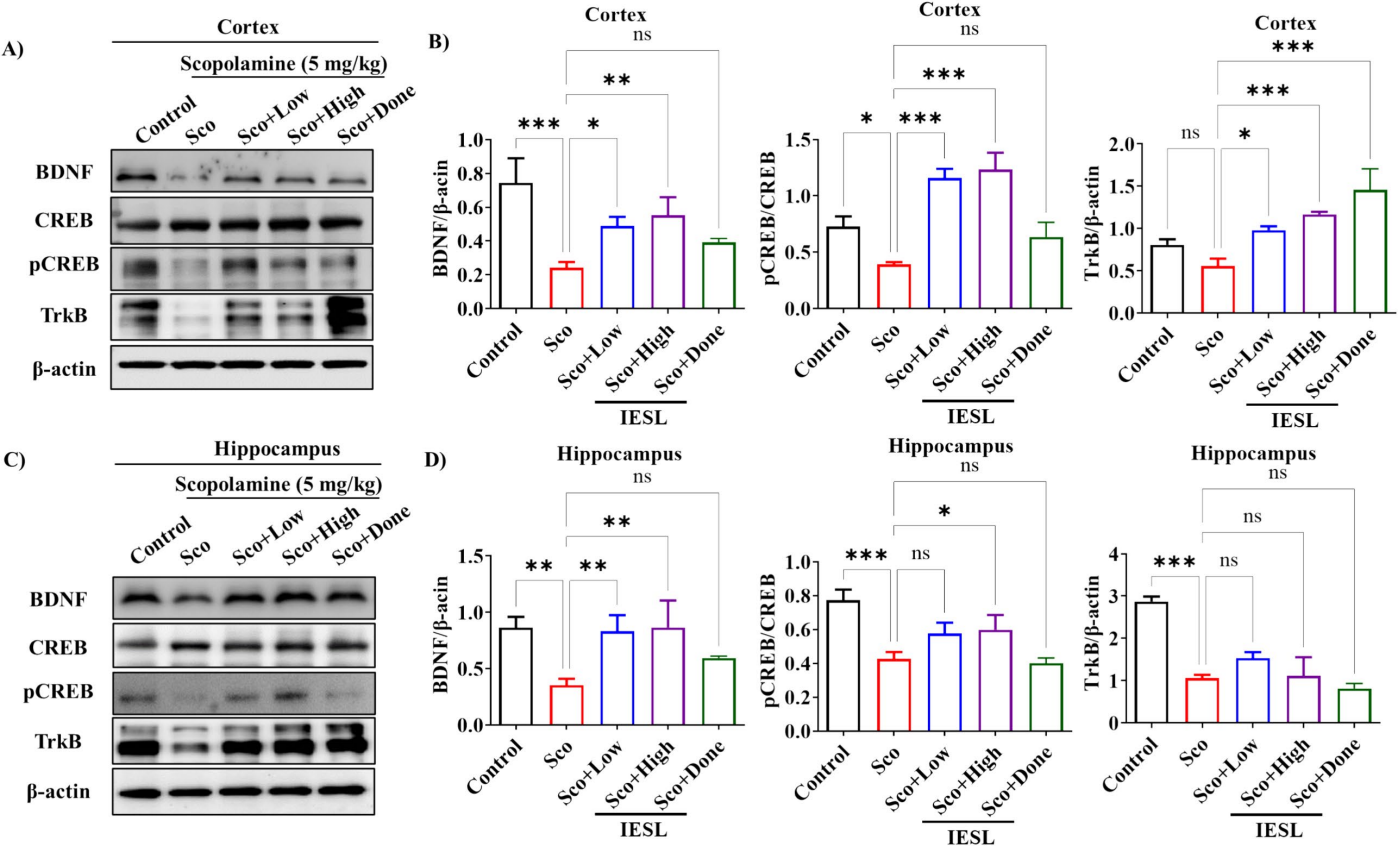
Effect of IESLs on CREB/BDNF signaling and associated neuronal proliferation and neurogenesis in the brain of scopolamine-induced mice. **(A)** BDNF, CREB, p-CREB, and TrkB protein expressions in the cortex (*n* = 3), **(C)** in the hippocampus (*n* = 3) were determined via western blotting and **(B,D)** represent the quantification of protein expression relative to *β*-actin or phosphorylated proteins using the ImageJ software. Data in the histogram are expressed as means ± SD of three independent experiments (*n* = 3 per group). ^*^p < 0.05, ^**^p < 0.01 and ^***^p < 0.001 vs. ontrol or Sco.

Another mechanism through which IESLs improve cognition and memory was investigated through a series of experiments analyzing BDNF expression (a neurotrophic factor) and the activity of upstream transcription factors (CREB and TrkB) in the cortex and hippocampus. In the cortex, scopolamine administration reduced BDNF protein expression, but this reduction was reversed in a dose-dependent by IESLs administration (Figure 5A). Meanwhile, various upstream transcription factors regulate BDNF expression, with CREB recently attracting particular attention (Sreelatha et al., 2024). CREB is a transcription factor that binds to the promoter regions of genes involved in memory and synaptic plasticity. Its activation induces the transcription of genes associated with memory formation and reinforcement. Intraperitoneal administration of scopolamine significantly reduced phosphorylated cAMP-response element-binding protein (pCREB) levels in the cortex, which was associated with memory impairment. However, IESLs administration and positive control group (Sco + Done), restored this to levels comparable to the control group, whereas TrkB expression also significantly increased (Figures 5A,B). Furthermore, scopolamine administration (SCO) significantly decreased BDNF expression in the hippocampus. However, IESLs treatment groups (Sco + Low or High doses) significantly increased BDNF levels, although only a slight increase was observed in the positive control group (Sco + Done). p-CREB levels, oral administration of IESLs and Sco + Done groups restored these levels similar to the control group, but TrkB expression remained unaffected (Figures 5C,D).

### Effects of IESLs on the apoptosis pathway in the brains of scopolamine-induced mice

**Figure 6:**
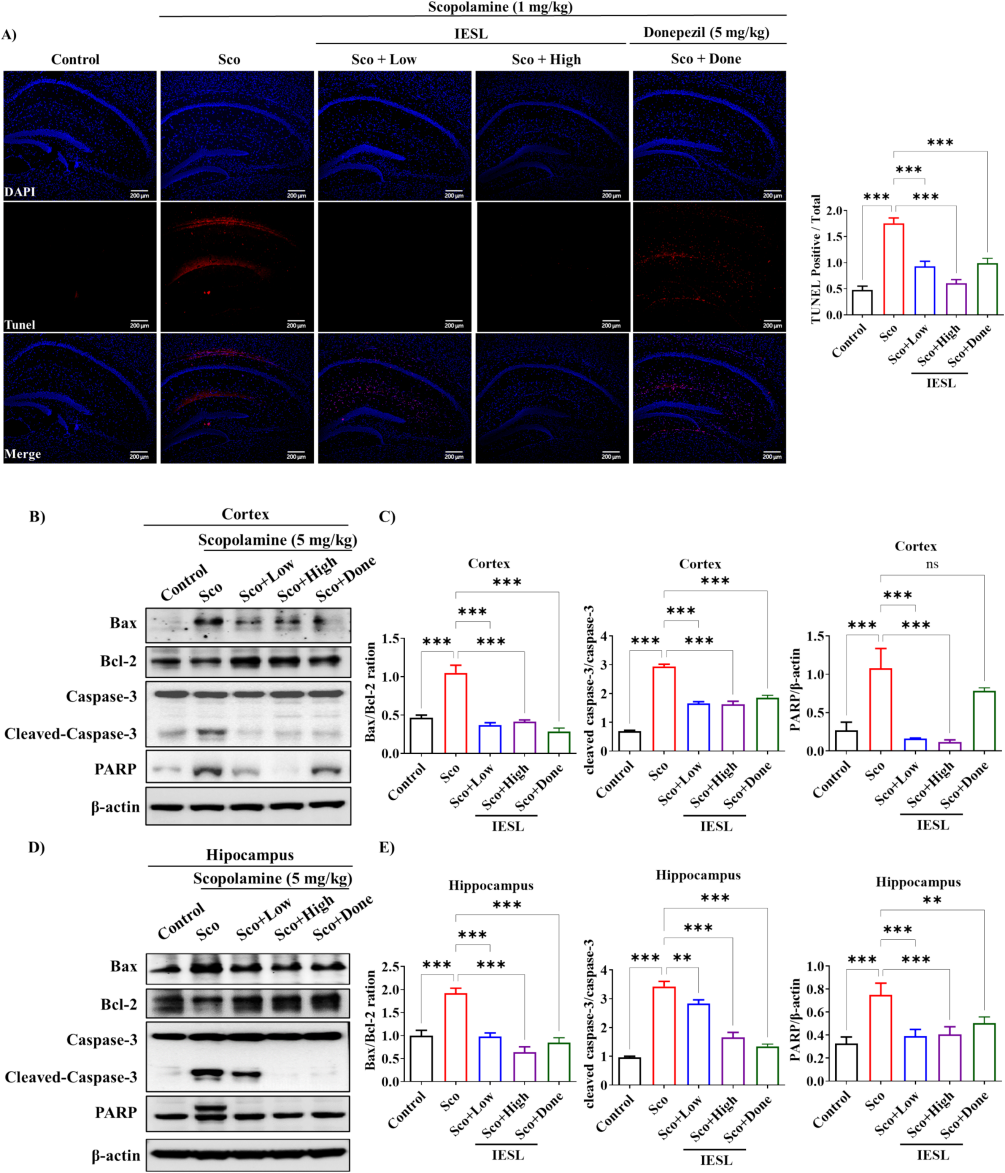
Effect of IESLs on the expression of apoptosis-related proteins in the brain of scopolamine-induced mice. **(A)** Histological sections represented TUNEL staining of the hippocampus and pictured under a fluorescent microscope at magnifications of ×100. White box was DAPI- and FITC-positive cells. **(B)** The supernatant of cortex and **(D)** hippocampus homogenate was subjected to SDS-PAGE, and western blotting analysis was performed using each specific antibody against Bax, Bcl-2, Caspase-3, Cleaved-caspase3, and PARP. β-Actin was used as the loading control. **(C,E)** Represent the quantification of protein expression relative to β-actin using the ImageJ software. Data in the histogram are expressed as means ± SD of three independent experiments (*n* = 3 per group). ^**^p < 0.01 and **^*^p < 0.001 vs. Control or Sco.

In scopolamine-induced mice, TUNEL and western blot analyses were performed on hippocampal tissue to confirm the protective effect of IESLs against apoptosis. As expected, scopolamine treatment significantly increased apoptosis, but IESLs effectively counteracted this effect (Figure 6A). Furthermore, the brain tissue was divided into cortex and hippocampus for apoptotic protein analysis results are shown in Figures 6B–E. The results showed that the scopolamine-treated group had a significant increase in the apoptotic proteins Bax and cleaved-caspase-3 along with a significant decrease in the antiapoptotic protein Bcl-2 compared with the control group (Figures 6B,D). However, in the IESLs-administered group, the downregulation of Bcl-2 expression and upregulation of Bax expression were prevented, thereby reducing apoptosis in the cortex (Figure 6C) and hippocampus (Figure 6E). In addition, the levels of cleaved caspase-3/caspase-3 ration and PARP protein expression was significantly up-regulated in the scopolamine-treated group compared with the control group (Figures 6B,D). However, the IESLs administration (Sco + Low, Sco + High) and Sco + Done groups showed lower levels of these apoptosis-related protein expressions in the cortex (Figure 6C) and hippocampus (Figure 6E).

### Effects of IESLs on oxidative stress in the brains of scopolamine-induced mice

**Figure 7:**
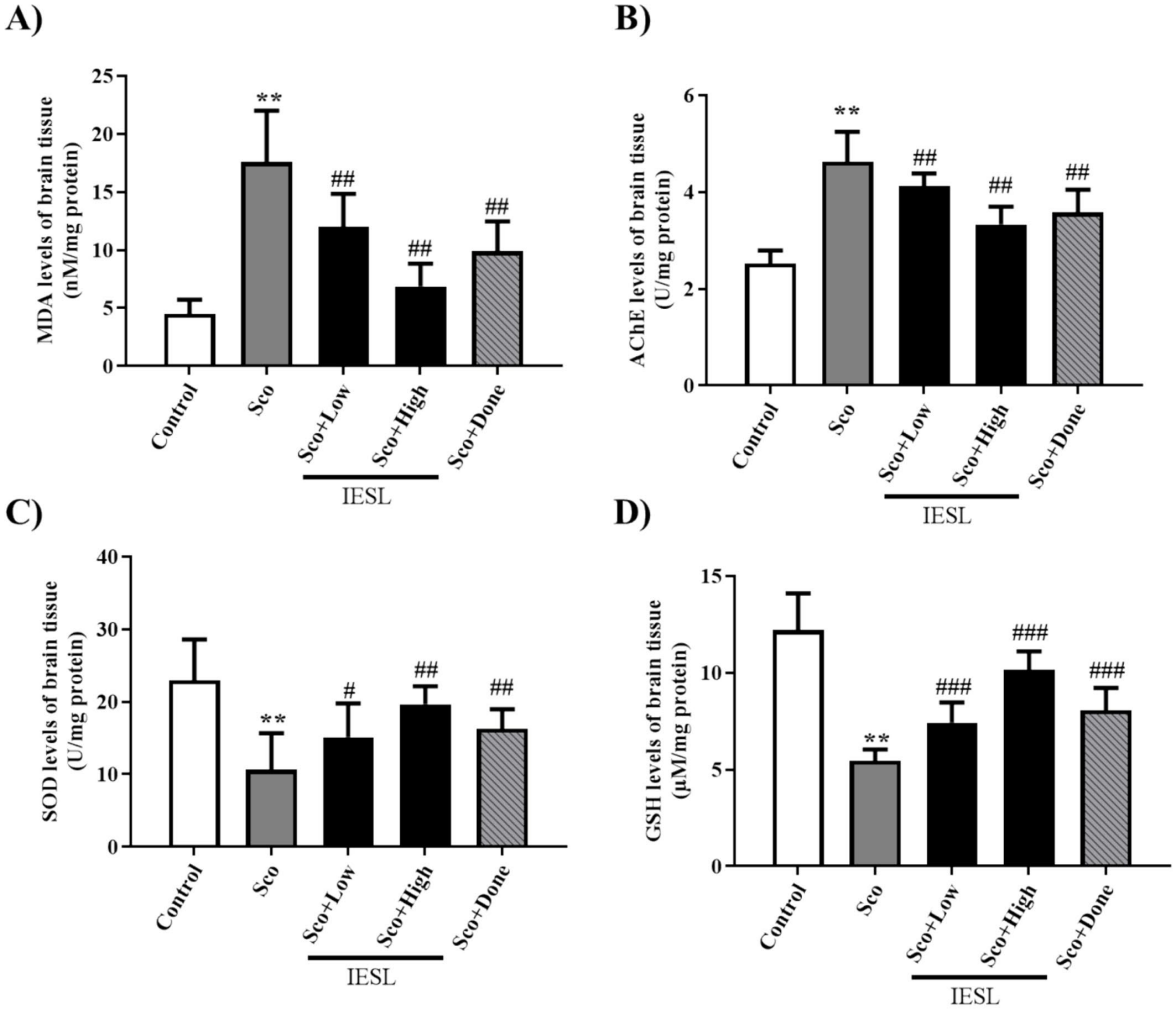
Effect of IESLs on the expression of oxidative stress in the hippocampus of scopolamine-induced mice. Graphs exhibit the **(A)** MDA level, **(B)** AChE activity, **(C)** SOD level, and **(D)** reduced GSH activity. Data are expressed as means ± SD of three independent experiments (*n* = 3 per group). ^**^p < 0.01, vs. Control; and ^#^*p* < 0.05, ^##^*p* < 0.01, and ^###^*p* < 0.001 vs. cSco.

To evaluate the effect of IESLs on oxidative stress markers, including lipid peroxidation and the antioxidant defense system in brain tissue, MDA and SOD levels and AChE and glutathione activities were measured. Compared with the control group, scopolamine-administered mice showed significantly elevated MDA and AChE levels (Figures 7A,B), whereas SOD activity and GSH levels were reduced (Figures 7C,D). However, the IESLs-treated group reversed these changes by restoring MDA content and AChE levels while dose-dependently preventing the decline in SOD activity and GSH content in brain tissue. Furthermore, the donepezil-treated group (Sco + Done) showed significant changes compared with the Sco group.

## Discussion

AD is characterized by generalized brain atrophy and specific histological features, including senile plaques, neurofibrillary tangles, and granulovascular degeneration of neurons (Ribeiro, 2023). Memory impairment, the primary symptom of AD, is directly or indirectly related to the dysfunction of the cholinergic neurons in the hippocampus and temporal lobe. In addition, biochemical dysfunction and neuronal loss in the cholinergic neurons of the basal forebrain pathway have been consistently reported in AD (Gedankien et al., 2023). Choline uptake and acetylcholine synthesis are reduced in the hippocampus and cerebral cortex of patients with AD, whereas the expression of acetylcholinesterase (AChE), the enzyme responsible for acetylcholine breakdown, is elevated. In addition, the activity of choline acetyltransferase (ChAT), an enzyme responsible for acetylcholine synthesis, and the number of nicotinic and/or muscarinic acetylcholine receptors (nAChR and/or mAChR) in the brain were reduced (Mohammad et al., 2017). Therefore, acetylcholine metabolism indicators are considered key factors in both the clinical monitoring of patients with AD and the development of therapeutic agents. Memory impairment is the most common and prominent symptom of dementia, significantly affecting patients’ social lives. Therefore, effective treatment for this symptom is crucial not only for alleviating symptoms but also for improving overall quality of life. In this study, we evaluated the efficacy of IESLs extract—an SL extract enriched with isoflavonoids—on memory impairment and cognitive function. This extract has previously demonstrated effectiveness in a menopausal dementia model (Xie et al., 2018). Therefore, in this study, to experimentally elucidate the cognitive and memory-improving effects of IESLs, scopolamine-induced memory impairment was established in C57BL/6 mice, and the protective effects of IESLs against memory impairment were evaluated. Scopolamine, the substance used in this study, is a muscarinic acetylcholine receptor antagonist that modulates acetylcholine activity without altering its concentration. As it produces electroencephalography (EEG) patterns resembling those seen in senile dementia and AD, it is being widely used to induce memory impairment models and advance the development of dementia prevention and treatment strategies (Thompson and Tobin, 2020).

In this study, various behavioral experiments, including the MWM and PA tests, were conducted. Cognitive and memory impairments induced by scopolamine were significantly alleviated following IESLs administration. In the MWM experiment, the scopolamine-administered group did not show a significant reduction in the time required to locate the hidden escape platform during the 5-day training period. In contrast, the IESLs-administered group showed improved memory over the training days compared to only scopolamine-administered group, allowing them to find the escape platform more quickly over time.

In the PA experiment, scopolamine-induced memory impairment caused experimental animals to forget the electric shock, resulting in shorter step-through latency when entering the dark chamber. In contrast, animals administered IESLs orally showed latencies comparable to those of the control group. According to a previous study, IESLs showed a protective effect against menopause-induced cognitive and memory impairment, as evidenced by improved spatial memory performance in the MWM test using a menopausal animal model. Furthermore, IESLs inhibited the activity of astrocytes and microglial cells, which mediate brain inflammation in the hippocampus and cortex (Xie et al., 2018).

To elucidate the mechanism by which IESLs improve cognition and memory, we conducted a series of experiments assessing oxidative damage and *in vivo* antioxidant defense mechanisms. Recently, since reactive oxygen species have been linked to various degenerative brain diseases, research on their in vivo generation and inhibition is being actively conducted (Yuk et al., 2016). Reactive oxygen species (ROS) are highly reactive, oxygen-derived molecules with greater oxidizing power than molecular oxygen (O₂). These include the superoxide anion, hydrogen peroxide, the hydroxyl radical, singlet oxygen, and the peroxy radical. ROS are generated by various enzymatic sources, such as the mitochondrial electron transport chain, cytochrome P450, NADPH oxidase, xanthine oxidase, and cyclooxygenase. Specifically, beta-amyloid—the causative agent of dementia—aggregates to generate reactive oxygen species. Conversely, artificially increasing antioxidant components and enzymes reduces beta-amyloid-induced cytotoxicity (Shen et al., 2024). In this study, scopolamine administration induced oxidative stress by increasing MDA production and AChE activity while decreasing endogenous antioxidants such as SOD and GSH, whereas these alterations were effectively attenuated by IESLs treatment. The body possesses a complex antioxidant defense network composed of enzymatic antioxidants (CAT, SOD, HO-1, and GCL), binding proteins, and low-molecular-weight antioxidants like GSH. Among them, GSH—a tripeptide molecule—plays a central role in maintaining redox homeostasis, detoxifying reactive oxygen species, and preventing lipid peroxidation (Chen et al., 2012). Hydroperoxyl radicals of lipids, formed by reaction with reactive oxygen species, abstract hydrogen atoms from the double bonds of adjacent unsaturated lipids, converting into hydroperoxides and alkyl radicals. When the alkyl radical combines with oxygen, it causes a chain oxidation reaction. IESLs demonstrated neuroprotective antioxidant efficacy, notably by upregulating the expression of SOD and GSH—key endogenous antioxidant enzymes (Anoush et al., 2022).

Recently, increasing attention has focused not only on enhancing antioxidant defense capacity but also on the role of various endogenous factors that protect neuronal cells. Among BDNF, a member of the neurotrophin family, plays a crucial role in promoting neuronal survival and synaptic function. It protects neurons from various neurotoxic factors and plays a crucial role in nerve development as well as in the maintenance of central and peripheral nervous system cells (Sreelatha et al., 2024). In the scopolamine-induced cognitive impairment model, IESLs treatment not only improved antioxidant capacity but also upregulated endogenous BDNF. Previous studies have shown that pilocarpine—a nonselective muscarinic acetylcholine receptor agonist—strengthens the cholinergic nervous system, increases BDNF mRNA expression in the hippocampus, and inhibits hippocampus-dependent memory impairment (Thomas et al., 2016). Transgenic mice with reduced expression of functional BDNF showed impaired synaptic transmission and long-term memory deficits (Gao et al., 2022). These impairments mirror the learning and memory dysfunction observed in patients with AD and severe cognitive impairment (Gao et al., 2022). Reduced expression of both BDNF precursor and mature protein was observed, demonstrating a negative correlation between memory impairment severity and BDNF expression levels (Sreelatha et al., 2024). Specifically, the substitution of valine with methionine in the BDNF single nucleotide polymorphism (SNP) results in the BDNFMet variant, which is associated with memory impairment and an increased risk of dementia, psychiatric disorders, Parkinson’s disease, depression, and bipolar disorder (Gao et al., 2022). Moreover, BDNF transmits signals through the TrkB receptor. When this signaling is impaired, the amyloidogenic pathway is activated, further increasing beta-amyloid production while reducing acetylcholine synthesis (Abdel-Lah et al., 2025). The cAMP-response element-binding protein (CREB) has been most strongly implicated as a transcription factor regulating BDNF expression. CREB plays a crucial role in neuronal survival, neuroprotection, and synaptic plasticity in degenerative brain diseases (Kim et al., 2022; Yue et al., 2025). It also regulates the expression of antiapoptotic proteins (e.g., Bcl-2), antioxidant enzymes, and BDNF (Park et al., 2024). In this study, IESLs increased BDNF expression by phosphorylating CREB in cortical and hippocampal tissues, thereby transmitting signals through the TrkB receptor. Moreover the significant increase in PARP expression observed in the scopolamine-treated group suggests the activation of DNA damage–related apoptotic pathways. Although PARP is essential for DNA repair, its excessive activation leads to NAD^+^ depletion and energy loss, ultimately causing neuronal damage. Thus, the elevated PARP levels are likely associated with oxidative stress and cognitive impairment induced by scopolamine (Kim et al., 2021). In contrast, the reduced PARP expression in the IESLs-treated group indicates a neuroprotective effect through the attenuation of oxidative stress and inhibition of apoptosis. These findings suggest that the compound may alleviate scopolamine-induced neurotoxicity and memory deficits by modulating the overactivated PARP pathway.

These findings collectively suggest that despite these promising findings, several limitations should be acknowledged in this study. First, the scopolamine-induced mouse model primarily represents acute and reversible cholinergic dysfunction rather than the chronic and progressive neurodegeneration observed in Alzheimer’s disease. Consequently, the findings of this study should be interpreted as focusing on cholinergic and oxidative stress-related cognitive impairment relevant to early-stage AD, rather than late-stage progressive neurodegeneration. Therefore, further studies employing chronic or transgenic AD models are required to confirm the long-term neuroprotective efficacy of IESLs. Second, this study was conducted using a single sex (male) and age group, without evaluating possible sex-dependent differences in response to treatment. Given that isoflavones function as phytoestrogens and can modulate estrogen receptor-mediated signaling, their efficacy and metabolic profile may vary significantly between sexes. Therefore, future research incorporating both male and female models, as well as considering hormonal fluctuations, is necessary to fully elucidate the sex-specific neuroprotective potential and clinical translational value of IESLs. Moreover, although IESLs treatment significantly enhanced antioxidant defense and upregulated the BDNF-CREB signaling pathway, mechanistic validation using specific pathway inhibitors (e.g., TrkB inhibitors such as ANA-12) or genetic modulation was not performed in this study. Therefore, further investigations utilizing these specific inhibition strategies will be necessary to definitively validate the therapeutic potential of IESLs and confirm the causal contribution of TrkB-mediated signaling to neuroprotection. Finally, long-term safety, pharmacokinetic, and toxicity assessments of IESLs are needed to ensure its translational potential for clinical application and Alzheimer’s disease models and pathway-specific inhibitors will be necessary to further validate the therapeutic potential of IES.

## Conclusion

This study demonstrated that IESLs protect neurons and improve cognitive function in a scopolamine-induced mouse model of cognitive impairment. These effects are mediated through enhanced cholinergic nervous system function, antioxidant activity, and associated behavioral and mechanistic changes.

## Data Availability

The original contributions presented in the study are included in the article/Supplementary material, further inquiries can be directed to the corresponding author.
