# Supplementary material for: Neuroprotective effects of isoflavone-enriched soybean leaves (Glycine Max) on scopolamine-induced memory deficits in C57BL/6 mice via antioxidative mechanisms
Source: Front Aging Neurosci. 2026 Apr 10;18:1787268. doi: 10.3389/fnagi.2026.1787268 (PMC13106398; doi:10.3389/fnagi.2026.1787268)
Supplement: Supplementary file 1 [file Data_Sheet_1.docx]

**Neuroprotective Effects of Isoflavone-Enriched Soybean Leaves (Glycine Max) on Scopolamine-Induced Memory Deficits in C57BL/6 Mice via Antioxidative Mechanisms**

Young Ju Do^1,2^, Seo Young Kim^2,3^, Younghoon Go^3^, Yong Hyun Lee^4^, Ki Hun Park^4^ and Tae Woo Oh^2,3*^

^1^ Department of Oriental Medicine Research Division, Korea Institute of Oriental Medicine (KIOM), Daejeon 34054, Republic of Korea

^2^ University of Science and Technology (UST), Korean Convergence Medicine Major KIOM, 1672 Yuseongdae-ro, Yuseong-gu, Daejeon 34054, Republic of Korea

^3^ Korean Medicine (KM) Application Center, Korea Institute of Oriental Medicine (KIOM), Dong-gu, Daegu 41062, Republic of Korea

^4^ Division of Applied Life Science (BK21 four), Institute of Agricultural and Life Science (IALS), Gyeongsang National University, Jinju 52828, Republic of Korea

***Corresponding authors:**

Tae Woo Oh, Ph.D., Korean Medicine (KM)-Application Center, Korea Institute of Oriental Medicine (KIOM), 70 Cheomdan-ro, Dong-gu, Daegu, 41062, Republic of Korea.

Tel: +82-53-940-3833; Fax: +82-53-940-3899, e-mail: taewoo2080@kiom.re.kr


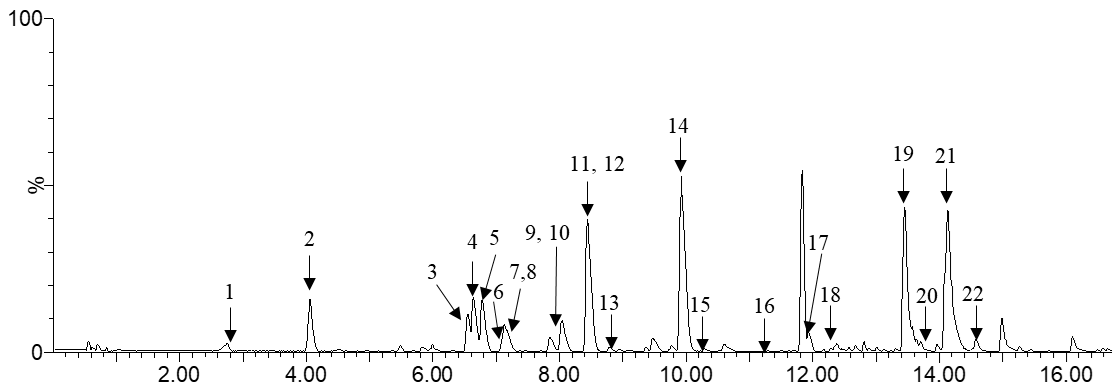


**Figure S1**. Base peak intensity (BPI) gram of IESL extracts by LC-Q-TOF/MS analysis. 1, phenylalanine fragment; 2, tryptophan fragment; 3, daidzin; 4, kaempferol-3-O-β-D-glucopyranosyl(1→2)-α-L-rhamnopyranosyl (1→6)-β-D-galactopyranoside; 5, kaempferol-3-O-β-D-galactopyranosyl (1→2)-α-L-rhamnopyranosyl (1→6)-β-D-glucopyranoside; 6, kaempferol-3-O-β-digalactopyranoside; 7, kaempferol-3-O-(2,6-di-O-α-L-rhamnopyranosyl)-β-D-galactopyranoside; 8, kaempferol-3-O-β-diglucopyranoside; 9, kaempferol-3-O-α-L-rhamnopyranosyl (1→6)-β-D-galactopyranoside; 10, genistin; 11, kaempferol-3-O-α-L-rhamnopyranosyl (1→6)-β-D-glucopyranoside;12, malonyldaidzin; 13, malonylglycitin; 14, malonylgenistin; 15, daidzein; 16, acetylgenistin; 17, formononetin 7-O-glucoside-6”-malonate; 18, genistein; 19, soyasaponin I; 20, soyasaponin III; 21, soyasaponin βg; 22, soyasaponin γg.


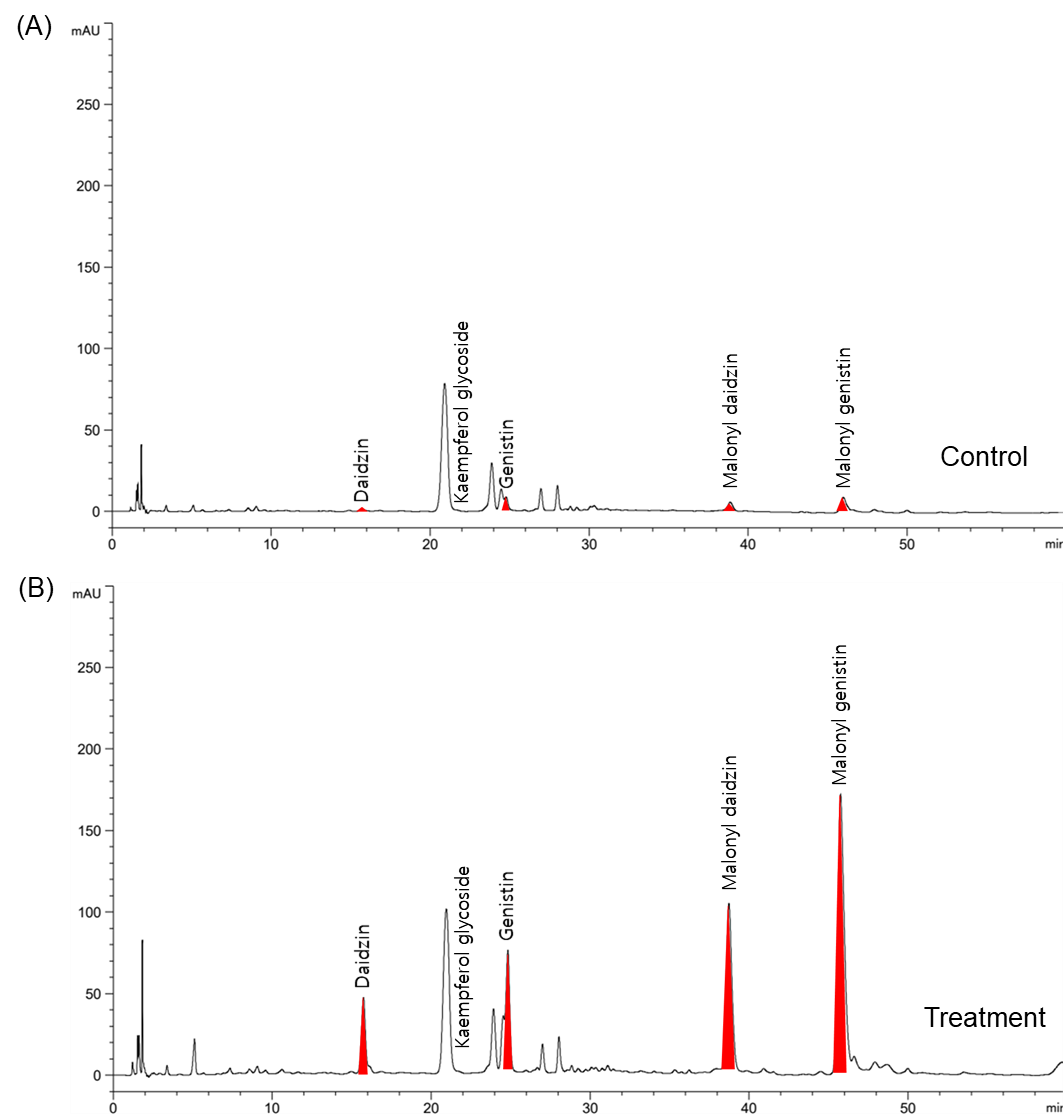
(A)

(B)

**Figure S2**. HPLC chromatogram of (A) normal soybean leaves and (B) isoflavone enriched soybean leaves extracts at 254 nm.
